# Tunable Interfacial to Filamentary Resistive Switching Mechanism in Room-Temperature-Grown Amorphous YBa_2_Cu_3_O_*x*_ with Excess Cu Addition

**DOI:** 10.1021/acsami.5c23772

**Published:** 2026-07-07

**Authors:** John Feighan, Ingon Kim, Ahmed Kursumovic, Tamsin Bedford, Abhijeet Choudhury, Juanjuan Lu, Sebastian C. Dixon, Haiyan Wang, Judith L. MacManus Driscoll, Markus Hellenbrand

**Affiliations:** † Department of Materials Science & Metallurgy, 2152 University of Cambridge, 27 Charles Babbage Road, Cambridge CB3 0FS, United Kingdom; ‡ School of Materials Engineering, Purdue University, Neil Armstrong Hall of Engineering, West Lafayette, Indiana 47907-2045, United States

**Keywords:** resistive switching, amorphous, oxide, YBCO, Cu, filamentary, nonfilamentary, area-dependent

## Abstract

Resistive switching technologies have the potential not only to create large efficiency gains in computer memory but also to revolutionize emerging fields such as neuromorphic computing. In this paper, we report on novel resistive switching behavior in devices made from room-temperature-grown Cu-rich amorphous YBa_2_Cu_3_O_*x*_ (YBCO) films, a material otherwise well-known as a high-temperature superconductor. In Nb:STO substrate/amorphous YBCO film (≈200 nm)/metallic Cu (15 nm)/metallic Pt (15 nm) devices, we demonstrate that the resistive switching can be tuned between mechanisms involving extended areas of the YBCO/electrode interface and a single-point filamentary mechanism simply by changing the Cu content of the deposition target and hence in the films. Changing the Cu content can also be used to optimize the properties of the devices further, with devices with an added 15 mol % of Cu in YBCO initially providing an on/off ratio >100, switching endurance potential >6500 cycles, and state retention >2 × 10^4^ s, all at low switching fields of 0.3 MV/cm. The amalgam of promising resistive switching properties, fast growth (150 nm/min) at room temperature, and tuneability of the switching mechanism indicates the strong potential of this proof-of-concept amorphous system for future memory applications.

## Graphical abstract

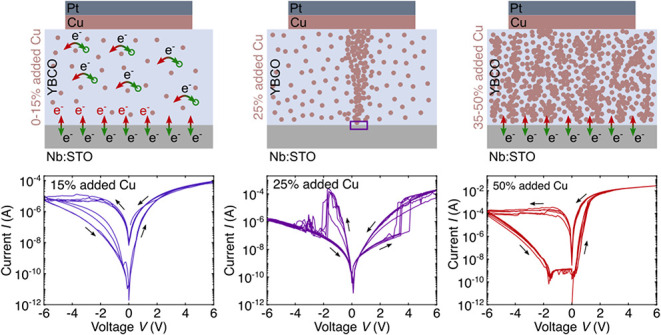

## 1 Introduction

As various forms of computing devices play an ever more important role in society, optimizing any aspect of data processing can lead to huge gains in both task execution time and energy efficiency. Memory is one such aspect whose speed and energy consumption can be improved via the development of new memory technologies. One of the current frontrunners for such a novel memory technology is resistive switching (RS) memory,^1^ where the resistance of a memory cell, consisting of two electrodes sandwiching a switching material, can be switched between a low resistance state (LRS) and high resistance state (HRS) via the application of an appropriate voltage. The fast switching, simple metal–insulator–metal structure, low power consumption, fast write/erase operation and nondestructive readout make resistive switching an ideal mechanism to replace many current memory architectures.^2^

Resistive switching properties have been found in a range of materials systems and with a range of switching mechanisms. They can be categorized into different groups such as area-dependent and -independent,^2^ or metal-ion-driven and oxygen-vacancy-driven, where the latter two mechanisms are often called electrochemical metallisation memory (ECM) and valence change memory (VCM), respectively.^3^ For ECM, upon voltage application, a filament can form from an active metal electrode such as Cu or Ag, and RS occurs by the repeatable formation and rupture of metal filaments. Due to the nanometer-small size of the filament, this mechanism is typically electrode-area-independent. VCM can exhibit area-dependent or -independent switching characteristics, but in both cases, the device resistance is typically determined by the height of a Schottky-like barrier between the switching material and one or both electrodes, and the height of the interface barrier can be tuned by the voltage-controlled oxygen vacancy concentration near the critical interface. In addition to a VCM mechanism, interface barrier height control can also be achieved by charge trapping and detrapping at the electrode interface.^4^

YBa_2_Cu_3_O_7‑δ_ (YBCO) is widely studied as a high-temperature (up to 93 K^5^) superconductor, but as a Cu-containing material, it could have great promise as a material for ECM, as the Cu supply required for the switching mechanism is not limited to the active metal electrode. Also, due to its remarkable superconducting properties, there is already widespread knowledge and expertise in producing YBCO films both in academia and industry,^6^ making the potential adoption of this material for memory devices significantly easier than many novel compounds. YBCO could provide a versatile materials system for example for cryogenic electronics, such as required for quantum computing, where the superconducting components could be realized in the crystalline, and the memory parts in the amorphous form of the same underlying materials system. However, studies of YBCO for RS are sporadic, missing typical RS performance metrics, and based on YBCO deposited at temperatures ≥750 °C, required for the superconducting YBCO properties, which are based on crystalline films.^7–9^ (See also Supporting Table S1.) Such high temperatures are incompatible with semiconductor industry fabrication requirements, and generally, amorphous thin films are preferred in industry. As well as needing less stringent deposition control, amorphous films can have depressed current leakage and greater uniformity due to the elimination of grain boundaries.^10^ For any industry relevance, it is critical for any RS materials system to be deposited at low temperatures. Here, materials deposition is carried out at room temperature, ensuring amorphous YBCO thin films and compatibility with a low temperature budget. The resulting devices demonstrate that by varying the Cu content in YBCO thin films by varying the Cu content in the deposition target, the RS properties of amorphous YBCO can be tuned across the entire range of YBCO-determined bulk switching, Cu filament switching, and interface-determined charge trapping.

Overall, the proof-of-concept amorphous YBCO system demonstrates the capability and versatility of amorphous, low-temperature-deposited YBCO with promising tunable properties such as HRS/LRS resistance ratios >100, potential for switching endurance of thousands of cycles, and state retention >2 × 10^4^ s. The work presents a strong starting point for further device development for versatile high performance nonvolatile memory devices.

## 2 Experimental Section

YBa_2_Cu_3_O_7_ + *x* at.% Cu pulsed laser deposition (PLD) targets with *x* = 0, 5, 15, 25, 35, or 50 were fabricated from powders of YBa_2_Cu_3_O_7_ and CuO via conventional solid state sintering (e.g., the 25% added Cu target had a molar ratio of YBa_2_Cu_3_O_7_ to CuO of 1:0.75, where 0.75 = 0.25 × 3 (Cu) for a total composition of YBa_2_Cu_3.75_O_*x*_). Amorphous films were deposited from these targets on commercial 5 × 5 mm^2^, 0.5-mm-thin (001)-oriented Nb-doped SrTiO_3_ (Nb:STO) substrates using PLD parameters of 50 Hz laser pulse frequency, 350 mJ laser energy, 200 mTorr oxygen pressure, 25 °C substrate temperature, 5000 pulses, and 4.5 cm substrate/target distance, resulting in a deposition rate of approximately 150 nm/min. The resulting films were between 150 and 250 nm thick, as confirmed by cross-sectional transmission electron microscopy (TEM, see later figure). A device consisting of Pt/Cu metal electrodes deposited directly onto the conductive Nb:STO substrate without YBCO film was studied as a reference. The samples are named after the added Cu amount in the target, i.e., films deposited from the YBa_2_Cu_3_O_7_ + *x*% Cu target are called *x*% samples.

Structural properties of the films, such as phase and crystallinity, were examined with X-ray diffraction (XRD, Figure S1 in the Supporting Information) to confirm that they were amorphous. To examine compositional and morphological features, energy-dispersive X-ray spectroscopy (EDX) measurements were carried out on the surface of the films and scanning TEM (STEM) and EDX was carried out on cross-sectional lamellas of two films with different Cu content (15% and 50% added Cu targets). X-ray photoelectron spectroscopy (XPS) analysis was carried out to reveal differences in the local chemical environment of Cu and O, and potentially to reveal the presence of oxygen vacancies by the associated Cu oxidation state, but the results were not conclusive. The associated challenges will be discussed.

To evaluate the electrical properties of the films, a series of contact electrodes on each film were deposited using standard UV lithography and sputtering. The electrodes consisted of bilayer Pt/Cu circles with diameters of 25–100 μm, creating a total device architecture (including approximate layer thicknesses) of Nb:STO/amorphous YBCO (≈200 nm)/metallic Cu (15 nm)/metallic Pt (15 nm). The approximate metal electrode thicknesses are derived from the sputter tool standard process parameters. Schematics of the device structure are provided in Figure 1. The current–voltage (*IV*) characteristics were measured using a computer-controlled^11^ Keysight B2912 source-measure unit together with a manual Everbeing Probe station, and the voltage was always applied to the Pt/Cu top electrode. For each Cu content, at least ten functional devices were measured to establish the switching pattern. Representative example *IV* curves for each Cu content are presented in Figure 1, and additional *IV* curves of multiple devices with different electrode sizes for the three key switching mechanisms are summarized in Supporting Figure S2. As the 15% samples performed the best overall, a second sample was fabricated and measured to demonstrate repeatability.

**Figure 1:**
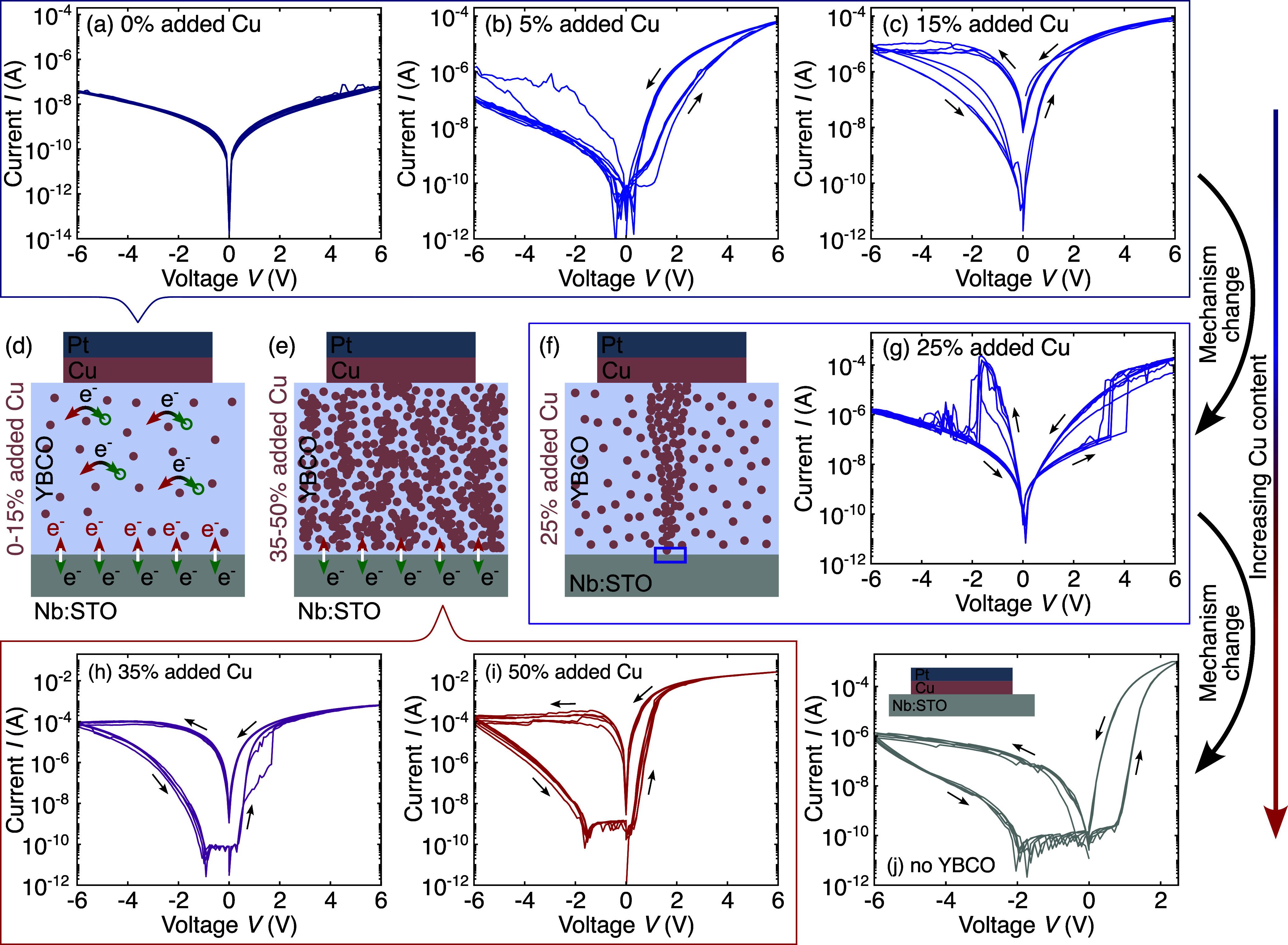
*IV* curves and switching mechanism schematics; black arrows indicate *IV* loop orientation. (a) Amorphous YBCO without added Cu acts as a low-conductance resistive element without switching. (b, c) For low amounts of added Cu in the target, area-dependent resistive switching behavior appears with an increasing hysteresis. (d–f) Schematics of the different observed switching mechanisms. (d) For 5–15% added Cu in the target, the switching is dominated by a Schottky barrier in one direction, and space-charge-limited conduction in the other, both controlled by charge trapping. (e) For 35–50% added Cu in the target, the YBCO film acts as a metallic contact, and switching occurs by charge trapping at the Nb:STO interface. (f, g) For 25% added Cu in the target, he switching is filamentary, revealed by the precipitous current jumps. (h, i) *IV* curves for 35–50% added Cu in the target. (j) Reference *IV* curve from a device consisting of Pt/Cu directly deposited on Nb:STO. Fits of Schottky emission and space-charge-limited conduction to the measured *IV* curves are presented in Supporting Figure S3.

Where data is presented as mean value and error bars, the error bars correspond to the standard deviation of multiple devices. Elsewise, the measurement error corresponds to the instrument noise as specified in the instrument manuals.

## 3 Results and Discussion

A lack of peaks in the *θ*/2*θ* XRD traces of the films in Figure S1 confirms that all films were deposited amorphously. The *IV* characteristics of the films are shown in Figure 1, together with switching mechanism annotations, which will be discussed in the following. The discussion starts with the lowest Cu content.

Devices which consist of amorphous films from targets with no added Cu (0%) act as insulators, resulting in no resistive switching effect and a high resistance, as shown by the lack of a hysteresis loop and low current flow in the *IV* plot in Figure 1(a). As the added Cu content increases gradually to 5% and 15%, Figure 1(b,c), respectively, the device currents increase and a hysteresis starts opening up, indicating the presence of resistive switching. At 25% added Cu, Figure 1(g), precipitous current jumps appear in the *IV* curves, which disappear again at the highest added Cu concentrations of 35% and 50%, Figure 1(h,i), respectively. The *IV* curve shapes of the latter two Cu concentrations resemble those of a reference sample, Figure 1(j), where the Cu top electrode was deposited directly onto the Nb:STO substrate. It is evident that with increasing Cu concentrations in the target composition, the electronic conduction mechanisms change, and alongside it, the resistive switching mechanism. To understand these better, the expected band structure across the device stack for low Cu concentrations, the scaling of the device currents with electrode diameter, and the state retention of the HRS and LRS were examined. The results are presented in Figure 2. Later on, this will be correlated with EDX and STEM experiments.

**Figure 2:**
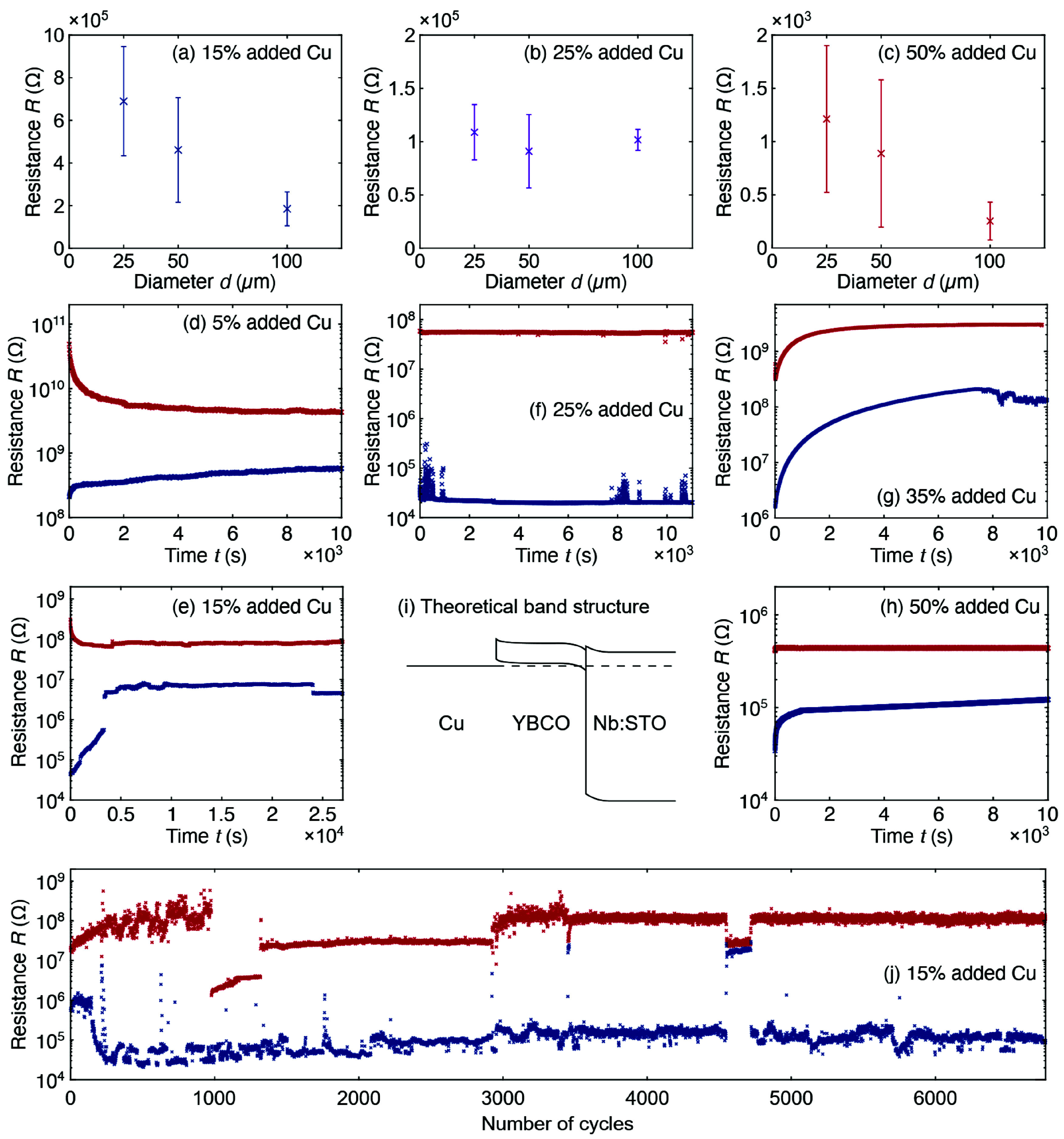
(a–c) Area dependence of the positive switching currents for 15%, 25%, and 50% added Cu samples. Five devices were measured per diameter per Cu concentration, and the mean and standard deviation are presented. (d–h) Retention measurements for all samples, measured at 1 V. (i) Band structure constructed according to the Schottky-Mott rule based on measured and literature values. (j) Switching endurance for the 15% added Cu samples.

Based on literature and measured values, the band structure of the Cu/YBCO/Nb:STO structure is expected to look like presented in Figure 2(i). The values for the work functions in this figure are Φ_Cu_ = 4.7 eV for Cu^12^ and Φ_YBCO_ = 4.6 eV for amorphous YBCO.^13^ Note that the work function for amorphous YBCO has been found to be considerably smaller than for crystalline YBCO, where values are typically 5–6 eV.^14^ Further for amorphous YBCO, a band gap *E*_g, YBCO_ = 0.46–1.3 eV has been reported, where the uncertainty occurs due to the abundant presence of localized states, e.g., from defects common in amorphous materials.^15^ As there are bound to be numerous defects in the amorphous YBCO here, we use the lower value of 0.46 eV. YBCO is typically a p-type material, and for its amorphous state, a carrier concentration *p* < 10^22^ cm^–3^ can be assumed,^16^ which places the Fermi level less than 0.1 eV underneath the valence band edge. (See Supporting Note 1 for details.) The values for Nb:STO were measured in our previous work as Φ_Nb:STO_ = 4.3 eV, *E*_g, Nb:STO_ = 3.3 eV, and the difference between the Fermi level and the conduction band as 0.3 eV.^17^

Based on these values, and a band alignment according to the Schottky-Mott rule, there is a near-ohmic contact between Nb:STO and YBCO, and a Schottky barrier of height ≈0.6 eV between Cu and YBCO. The near-ohmic contact is different from the interface between Nb:STO and crystalline YBCO, which has been reported as Schottky-like,^18,19^ and this is a result of the much smaller work function of amorphous YBCO as compared with its crystalline form. Based on this band alignment, for nonfilamentary switching, the likely electronic conduction mechanism in the forward direction (positive voltage at Cu electrode) is space-charge-limited conduction (SCLC).^20^ In the reverse direction (negative voltage at Cu), a barrier-limited electronic conduction process, such as a reverse-biased Schottky barrier,^21^ is likely, although SCLC can occur in the presence of a nonohmic contact, too.^20^ Each electronic conduction mechanism will be limited by a corresponding switching mechanism to control the current-limiting aspect of the band structure. Figure S3.1 confirms that indeed, for the lowest three additional Cu concentrations of 0%, 5%, and 15%, the expected electronic conduction models (SCLC forward, Schottky reverse) can be fitted reasonably well. It should be pointed out, however, that the fits are sometimes ambiguous, which could indicate a mixture of contributing conduction mechanisms such as Schottky emission and SCLC at the same time, and this is further complicated by the changing Cu content. As a rough quantification, with details provided in the Supporting Information, the calculated Schottky barrier heights from the fits in the negative voltage ranges of the 0–15% added Cu samples are between 0.61 and 0.85 eV for the LRS and HRS, respectively. This is in line with the values expected from the band diagram, where an equilibrium barrier height of 0.6 eV was estimated. In addition to the consideration of the band structure, the state retention and area dependence of switching currents at these Cu concentrations help elucidate the potential switching mechanism.

At 15% additional Cu, Figure 2(a), the average resistance of the devices changes with their TE area. This makes a filamentary process unlikely. The state retention in Figure 2(d,e) approximately follows a power law, which can be associated with a charge redistribution process.^22^ Altogether—SCLC and Schottky fitting, resistance area dependence, and charge redistribution—can be explained consistently by charge trapping as the underlying switching mechanism. During positive voltage application at the Cu electrode, electrons start populating traps in the YBCO bulk, while being depleted from interface traps between the YBCO and the Cu. This causes the device to switch from the HRS to the LRS, where for positive bias, the YBCO bulk contribution is current-limiting and thus determines the resistance state. This is in line with the different slopes of the fitted SCLC model in Figure S3.1, where the large slopes of the HRS branch indicate a trap-filling-limited behavior and the slopes of the LRS branches are lower, indicative of a reduction of this effect.^23^ Afterward, for a negative bias at the Cu electrode, the reverse-biased Schottky barrier between Cu and YBCO becomes the current-limiting element. The currents in this bias direction are smaller than in the forward direction, supporting the conclusion that a different element is current-limiting here. As the interface traps have been depleted of electrons during the positive voltage application, this barrier is at its lowest and the device is initially in the LRS also for negative bias at the Cu electrode. With increasingly negative bias, the interface and near-interface traps get populated again by electrons from the Cu side and the device returns to the HRS. This combined process is illustrated schematically in Figure 1(d).

As the different films, deposited with different added Cu concentration in the target, exhibit different conduction and switching mechanisms, it can be concluded that the structural and compositional change in the films, induced by the changing Cu target composition, is instrumental in controlling the amount of bulk and interface trapping. It should be noted that while the Cu concentration in the deposition target is the control parameter to tune the composition and structure of the thin films, the charge trapping itself at this target Cu content of additional 15% most likely occurs via oxygen vacancies (in the bulk) and interface defects (oxygen vacancies, metal-induced gap states, defective bonds), which are established trapping sites in oxides and at interfaces.^24^ The retention characteristics are then determined by the changed trap populations returning to their equilibrium states.^25^ For added Cu contents beyond 15%, the contribution of bulk oxygen vacancies diminishes, as discussed below.

At 25% additional Cu, Figure 1(g), the behavior of the devices changes drastically. The shape of the *IV* curves now includes a precipitous current increase/decrease at about 3–4 V and −2 V, a typical indicator of a bipolar filamentary resistive switching mechanism, schematically illustrated in Figure 1(f). Remarkably, the devices are self-compliant at currents <1 mA and do not require an external compliance current, different from many other filamentary devices in the literature.^26^ As presented in Figure 2(b), the device switching currents are independent of the device area, and this is another common observation for filamentary devices. The current branches in the HRS and LRS can all be fitted well by the Schottky emission model, shown in Figure S3.2, which is further in line with previous reports of filamentary switching.^27^ The fitted value of the Schottky barrier height is ≈0.30 eV in the HRS and ≈0.24 eV in the LRS, which is close to the distance between the Fermi level and the Nb:STO conduction band. Once programmed, the HRS and LRS remain stable with an LRS/HRS current ration of 3 orders of magnitude, see Figure 2(f). Based on all of these electrical signatures—precipitous current changes, Schottky barrier height, device area independence, and stable retention—it can be inferred that the switching mechanism at 25% added Cu is based on a Cu filament, extending from the Cu TE toward the Nb:STO substrate, and the current-limiting Schottky barrier appears between the Cu filament and the Nb:STO. In oxides in general, filaments are often based on oxygen vacancies, but in Cu-containing systems, they are typically composed of Cu. (See e.g., references in Supporting Table S1.) Some fluctuations are apparent in the LRS for *t* < 1 × 10^3^ and *t* > 8 × 10^3^ s, and they are likely stochastic fluctuations in the state of the filament, e.g., some stochastic atomic reconfiguration. The large overall memory window, typical for filamentary devices, keeps these fluctuations contained and would still be suitable for memory.

At 35% and 50% additional Cu, this filamentary behavior disappears, though it should be noted that a minority of devices at 35% did show unstable filamentary behavior, with initially filamentary-shaped *IV* curves morphing into smoothly shaped *IV* curves after three cycles or less—see Supporting Figure S4. While this initial filamentary behavior is not stable, it contributes to understanding the switching mechanism in these devices. Together with the observation that the shape of the *IV* curves at these Cu concentrations is very similar to those of film-free Pt/Cu/Nb:STO devices, Figure 1(j), and furthermore to literature observations of metal directly on top of Nb:STO,^4^ it stands to reason that in these devices with the highest additional Cu concentrations, the YBCO film simply acts as a conductive interlayer, and the resistive switching is again dominated by charge trapping at the interface. This is illustrated schematically in Figure 1(e) and has been discussed in detail in the literature.^4^ The electrode area dependence and state decay in Figure 2(c,g,h) are in line with this interpretation. The striking resemblance of the *IV* curves, including almost identical switching voltages and current levels, and the charge detrapping state decay, lead to the conclusion that the Cu-rich YBCO provides a matrix for very conductive, Cu-rich paths, probably metallic, and this is strongly supported by the TEM and EDX results discussed later. Finally, at these highest added Cu concentration, the *IV* curves can be fitted well with a reverse-biased Schottky emission model, consistent with a contact between a highly conductive material (Cu-rich films) and a semiconductor (Nb:STO). It should be noted that the SCLC model can be fitted similarly well as the Schottky emission model, which may indicate that there are competing electronic conduction mechanisms at play, such as a competing element of the SCLC bulk in series with the interface trap switching. The fits are presented in Supporting Figure S3.2.

While all devices, after an initial decay, maintained stable memory windows for several 1000 s, the 15% devices additionally demonstrated a strong resilience to decay over 6500 switching cycles, Figure 2(j). On the other hand, the filamentary 25% devices had relatively poor endurance, with a LRS breakdown after ≈100 cycles. (Data not shown due to poor performance.) Such a low endurance seems characteristic of Cu-containing filamentary devices: With one notable exception, switching endurances are reported with only a few hundred cycles, up to 1000, see Supporting Table S1 for a literature overview. Here, while not perfectly stable yet, >6500 reported cycles constitute a strong improvement, and are expected to be far from the breakdown limit, with promise for further improvement in the future. With the superior endurance (relative to the other Cu concentrations) and all combined metrics, the devices made from the 15% films had the best overall properties. A concise comparison of all films in this work, together with the comparison with literature reports, can be found in the aforementioned Supporting Table S1.

The Cu-based switching in Cu-rich films also enables low switching fields, with a maximum of 0.3 MV/cm for the nonfilamentary devices, and as low as 0.1 MV/cm for the filamentary ones, in line with literature results on Cu-rich films (see Supporting Table S1), and much lower than typical non-Cu-oxide-based devices or entirely Cu-free systems. With some examples of devices with Cu electrodes but non-Cu oxides provided in Supporting Tabe SI, the switching fields quickly rise to 0.5–1 MV/cm and above, and many entirely Cu-free devices, e.g., based on hafnium oxide as a widely researched resistive switching material, require switching fields on the order of 1 MV/cm or higher.^26^

Finally, close examination of the 15% retention characteristics indicates that there may be an onset of combined area and filament switching characteristics, as the 15% state retention exhibits some step-like features and after an initial decay, is unusually stable for nonfilamentary devices.^26^ Similar “hybrid switching” characteristics have been demonstrated in other materials systems^17,28,29^ and importantly, unlike many conventional area-dependent VCM devices which employ materials bilayers,^30^ this can be achieved in single-material device stacks, which provides the great benefit of easier manufacture.

Overall, the dependence of the LRS/HRS conduction ratio, loop shape, and change in dominant resistive switching mechanism with the target Cu content indicates that the target Cu concentration provides a strong control variable for establishing the right materials conditions for different mechanisms. This does not rule out that oxygen migration also plays a role in some of the switching mechanisms, but the strong Cu content dependence indicates that its concentration is a dominating factor and can be used to tune the overall thin film composition and structure.

The translation of the target Cu content to the thin films was examined by EDX, scanning the top of the thin films perpendicularly to the surface in a scanning electron microscope (SEM). The elemental maps are provided in Supporting Information Figure S5 and the extracted full-area Cu contents, normalized to the Y content, are summarized in Table 1. (The 50% added Cu samples is not included in the EDX analysis because it was damaged in the preceding XPS experiments, discussed below.) The Supporting Information also contains additional measurements of the composition at selected spots, free from any morphological features in the SEM scan, to check whether such morphological features are potentially formed by local compositional variations. The full-area map and spot measurements, with values for the latter provided in Supporting Table S2, provided the same composition results within the accuracy of the measurement, and, anticipating the following discussion, it is clear that increasing Cu contents in the PLD target lead to increasing Cu contents in the thin films. It should be noted that while the oxygen EDX maps are provided for reference, and to illustrate qualitatively the uniform oxygen distribution across the films, the oxygen content cannot be quantified reliably by EDX, among other reasons because the weak emitted oxygen X-rays get absorbed by the specimen itself.^31^ Different from the Cu content, the Ba content, normalized to the Y content, did not show any trend, and yielded Ba/Y ratios of 1.4–1.8. (See Supporting Table S2.) This is expected as Ba loss during PLD of YBCO is commonly observed,^32,33^ and the fluctuations are well within the commonly accepted accuracy limits of standard EDX mapping.^31^

**Table 1:** Added Cu Content and Nominal Composition in the PLD Targets, and Measured Cation Ratios in the Thin Films from EDX Elemental Area Maps

Added Cu, target	Cu/Y ratio target, nominal	Cu/Y ratio EDX
0%	3	3.93
5%	3.15	4.44
15%	3.45	4.90
25%	3.75	5.23
35%	4.05	6.29

It is remarkable, however, that the measured Cu/Y ratio in the films is much higher than in the target. The two obvious potential reasons could be truly a high Cu concentration in the films, or a systematic offset in the EDX measurement. The results of EDX composition measurements typically fall within a ±25% accuracy range;^31^ the observed compositions are marginally outside this range. If indeed the Cu/Y ratio is strongly elevated, this change versus the target composition must occur due to deposition parameters different from the typical high-temperature epitaxial process. This is because the 0% reference YBCO target used here is the same type of target that gives near stoichiometric or slightly Cu-reduced^33^ stoichiometries in high-temperature depositions. The two key different deposition parameters here are the high laser frequency of 50 Hz, 4–60 times as high as typical YBCO epitaxy,^34^ and the room temperature deposition as opposed to typical 750–850 °C substrate temperatures.^34^ Two consequences of these differences could be local composition changes in the target during ablation, or different adsorption/re-evaporation contributions from the substrate, respectively. Given that copper oxide has the lowest melting point compared with yttrium oxide and barium oxide, intense laser exposure and heating could lead to increased or prolonged melting of the Cu phase and cause preferential ablation of Cu-containing species and hence an increased Cu concentration in the film. Additionally, at high substrate temperatures, excess Cu may re-evaporate from the substrate^35^ if not incorporated into the film structure, whereas this would be strongly suppressed with a substrate at room temperature, and could add to the increased Cu content. However, these are only initial potential explanations, and detailed follow-up studies would be required to resolve the reasons accurately. Either way, the important confirmation for the purpose of this work is the increased Cu content in the thin films as a result of increased Cu contents in the PLD target.

For two selected samples with strongly different nonfilamentary RS behavior, 15% and 50% added Cu in the target, the SEM EDX was complemented with cross-sectional TEM and EDX across the lamella. The results are presented in Figure 3. First, it is evident that both films are very dense and while structural features are visible, these are distributed evenly throughout the film. The high density is remarkable given the room temperature deposition of the thin films. Given that two examples with very different Cu target concentrations both show dense, uniform films, the other films can be expected to appear similar. As before, the main differences in composition and structure are related to the Cu concentration, which are Cu/Y ratios of 3.15 and 4.39 for the 15% and 50% target, respectively. While the difference in Cu concentration is evident here, too, the numbers themselves differ between SEM and TEM EDX. The reasons can again be twofold. Either there is a difference in measurement sensitivity/accuracy, or the difference in Cu/Y ratio reflects a difference in large-area averaging (SEM EDX) and focused local composition (TEM EDX). The measured Ba/Y ratios were 1.80 and 0.32 for the 15% and 50% target samples, respectively. While the 15% sample Ba/Y ratio is in line with the other samples, for the 50% sample, it is remarkable that the already-mentioned Ba loss is especially pronounced, even though it is of yet unclear why. It could again be a local variation revealed by the local EDX probing in the TEM lamella, or indeed a critical change in the overall film composition due to the very high Cu content. Additional thin films with target compositions between 35% and 50% added Cu could be studied to resolve the onset of such a drastic change.

**Figure 3:**
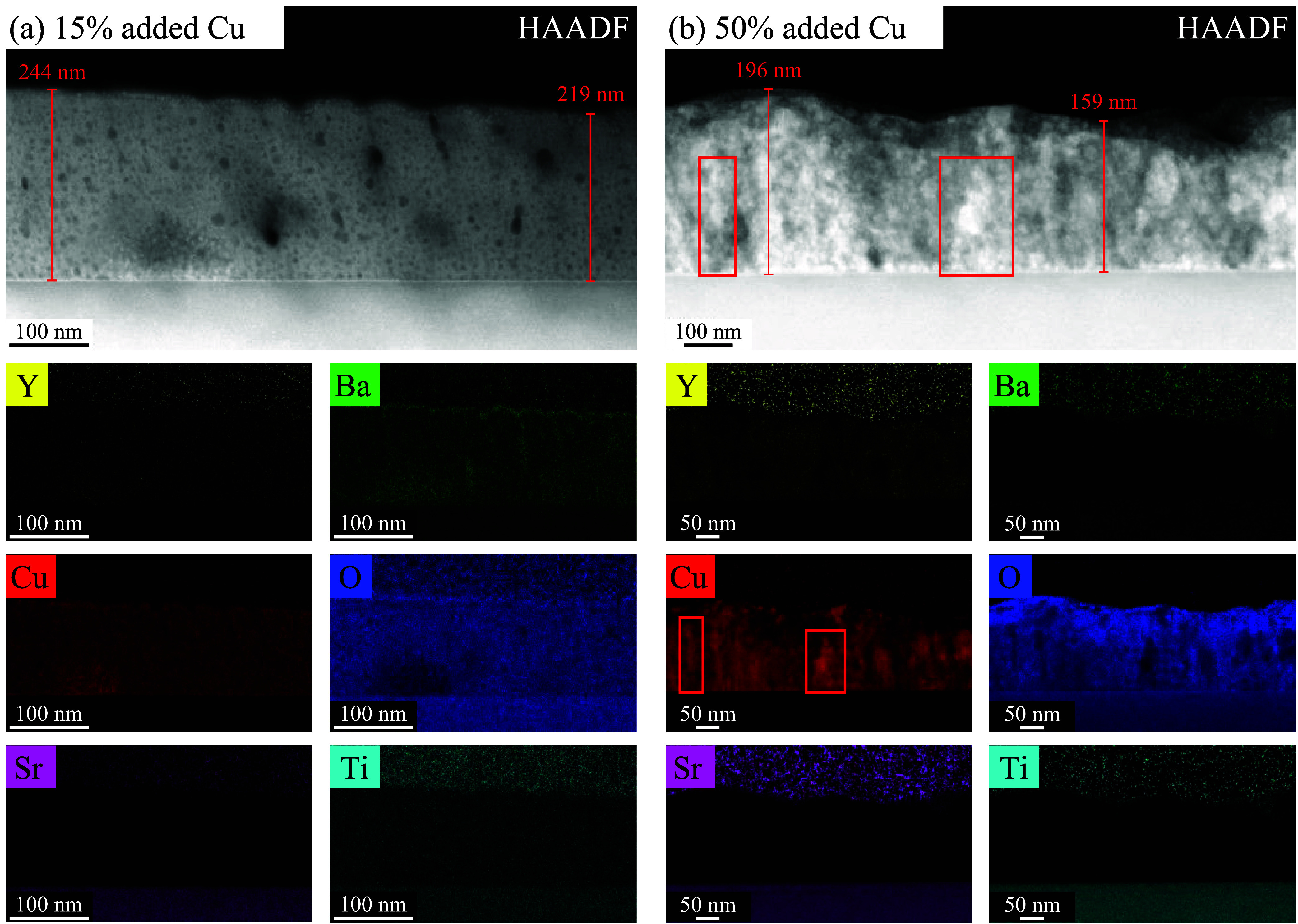
Scanning transmission electron micrographs (STEM) and energy-dispersive X-ray spectroscopy maps of two thin films deposited from (a) 15% added Cu target, (b) 50% added Cu target. Top two images: STEM in high-angle-annular dark-field mode; EDX maps below for each film. Note that the oxygen map cannot provide accurate quantification and is only provided for reference. The Nb map is not included as Nb as a dopant only has a very weak signal. The oxygen signal outside of the sample area is likely mostly from the TEM lamella, and for the other elements they are measurement artifacts due to integration.

In addition to the overall difference in Cu content, the Cu is uniformly distributed throughout the 15% film, but cluster-like structures are visible throughout the 50% film. One example of such a cluster is marked in Figure 3(b) for orientation, and Supporting Figure S6 provides a high-resolution image of some of the clusters. Some of these structures extend almost through the entire film, and, together with the overall high Cu concentration, can explain the near-metallic behavior of these films, leading to interface switching at the Nb:STO electrode. The uniform distribution in the 15% films on the other hand provides a uniform structure for area switching. Even though the oxygen content cannot be measured accurately, it stands to expect that a change in the cation ratio will be accompanied by a change in the oxygen content, and since oxygen vacancies are known to play an important role in RS devices,^36,37^ this change will likely affect the investigated devices, too. The controlled parameter in this work, however, is the target Cu content, and so the observed change in RS in the various films is ultimately induced by changing the Cu content of the deposition target.

As before, the oxygen EDX maps are provided for reference, and to illustrate qualitatively the oxygen distribution throughout the films, but the oxygen content cannot be quantified for the reasons explained above. Furthermore, there is a lot of background signal from oxygen adsorption on the TEM lamella during sample preparation, confounding the oxygen EDX signal further. For an accurate measurement of the oxygen concentration, EDX would have to be combined with other methods such as energy electron loss spectroscopy (EELS) or elastic recoil detection analysis (ERDA), but both are beyond this initial study. For the heavier cations in the films, EDX provides reliable results.^31^

Another technique commonly used to attempt to quantify elemental compositions and the presence of oxygen vacancies is XPS. Despite an increasing volume of cautionary literature about the proper use of this technique,^38–40^ it is still widely used for analysis in oversimplified manners in other fields. Accurate quantification of complex multielement materials such as amorphous YBCO warrant their own dedicated publications. We attempted to employ XPS to gather at least potentially supporting qualitative information about our YBCO thin films; the following insights and challenges can be summarized.

XPS is a surface analysis technique, and so depth profiling by sputter etching would be required to provide any meaningful compositional information. However, sputter etching induces changes to the core levels themselves,^41^ and would hence require the careful design of reference experiments to distinguish between sputter damage and actual thin film properties. Additionally, in a multielement material like YBCO, preferential sputtering of Cu would be expected due to the large mass difference between the three cations,^42^ and so sputtering would not only change the chemical states, but also the composition itself.

In oxide RS devices, oxygen vacancies are often involved in the switching process,^36,37^ and so their presence and charge states are of importance. Since oxygen vacancies cannot emit any spectroscopic signals,^40^ their presence is often inferred from different oxidation states of associated cations.^40^ In the case of YBCO, this would be Cu, which can occur as Cu^1+^ or Cu^2+^. Y and Ba ions typically only occur as Y^3+^ and Ba^2+^, and their enthalpy of formation is much more negative than for Cu^*x*+^, so that the presence of Cu^1+^ would be the best indicator of oxygen vacancies. The surface XPS Cu measurement, Supporting Figure S7­(a), reveals the strong presence of Cu^2+^, identifiable by their characteristic satellites, underneath which the potential Cu^1+^ satellite cannot be identified.^43^ The main 2p_3/2_ peaks appear shifted for some of the added Cu concentrations, which could indicate a difference between Cu^2+^ and Cu^1+^. However, it is not clear whether the difference is a true chemical shift or just related to sample charging, as the peak positions of the different measurements cannot be compensated reliably. The employed XPS tool required Cu clasps to affix and ground the samples, but obviously, the Cu reference cannot be used to correct for peak shifts here. The C 1s peak is still commonly used as a reference in literature, although this practice has been strongly condemned as inaccurate.^38^ It would not have been possible here, anyway, because the C 1s peak appears in a complex convoluted shape itself (Supporting Figure S7­(c)). In addition to the carbonaceous adsorbates present on any sample, this could be for example because Ba very easily reacts to form additional carbonates.^44^ For similar reasons of impossible peak calibration, the shifts in the O 1s signal (Supporting Figure S11­(b)) cannot be used to reveal the presence of oxygen vacancies. In future work, this could be addressed by designing a new set of samples, e.g., with additional Au contacts close to the measured sample area and connecting the samples to the instrument, to be able to calibrate any peak shifts.

To confirm the aforementioned challenge of depth-profiling this complex material by sputter etching, cation ratio quantification of the composition after 600 s etching with Ar^+^ ions was carried out, and the results are summarized in Supporting Table S2. It is clear that the Cu content from this measurement is much lower than both the EDX results and the target composition, which is readily explained with the preferential removal of Cu as the lightest cation in the films. The Ba content stays approximately the same for all samples, and the Ba/Y ratio, also without systematic change, is consistent with the EDX results.

Overall, the observed RS behavior for different Cu content in amorphous YBCO reveals a notable discovery—that the switching mechanism can be changed simply by controlling the target Cu content, moving between area-controlled and filamentary. This is especially clear between the 15% and 25% films, and then again between the 25% and 35% films. The ability to produce a filamentary switching mechanism by changing the Cu content of the films also indicates that the filaments consist of Cu channels. The mechanism for this is shown in schematic form in Figure 1 alongside schematics for the YBCO-bulk- and Nb:STO-interface-limited switching. The lack of filamentary behavior for the amorphous films with low additional Cu (0%, 5%, and 15%) is consistent with the Cu ion diffusion distances being too large to form a connected filament. At the same time, low amounts of added Cu (5% and 15%) provide levels of electronic conductivity and disorder which enable a nonfilamentary resistive switching effect. At 15% added Cu, this balance reaches an optimum, with a sizable memory window (missing at 5%) and promising endurance and retention. Combined, the demonstrated performance is the best among the compositions in this work, with a summary of metrics in Supporting Table S1. For Cu contents of about 25%, filamentary connection is much easier as the Cu ion diffusion distance is reduced. For >25% added Cu, the films can become saturated with highly conductive pathways, e.g., forming multiple unbreakable filaments, or become bulk-conducting, and then the films act as a low-resistance series element, almost like an extension of the electrode. This is confirmed by the *IV* loops for the 35% and 50% films being similar to the Nb:STO control sample, and by the STEM results, which reveal Cu-rich clusters extending through large areas of the film.

The fact that the mechanism for resistive switching can be altered very simply by changing the Cu amount in the targets permits a single materials system to exploit the advantages of an area switching mechanism (endurance) or a filamentary mechanism (retention, speed), depending on the required functionality. The entire pathway between the change in target Cu content and final film composition is not understood at the atomistic level yet, but this initial work highlights the promise of the YBCO materials system. It can also be a great benefit in large-scale fabrication, as it only requires changes in composition as opposed to changing a complete set of precursor materials, and industry-scale YBCO fabrication (e.g., for superconducting tape) already exists. Compared with other Cu-based amorphous switching devices, such as Cu-doped silicon dioxide films where two fabrication steps are necessary (one SiO_2_ deposition step, then a second thermal treatment with a Cu source or ion implantation), here only a single deposition step is required, speeding up the production, improving the uniformity, and decreasing the likelihood of damage to periphery electronics. There is also the potential advantage (to be explored in follow-on studies) that since the Cu ions are inherent to the YBCO composition and are distributed evenly throughout the thickness, compared with ion-implanted Cu filamentary materials systems, there can be smaller ion diffusion distances, and thus likely faster switching speeds. Potentially, the rich presence of Cu in the films near the critical switching sites and the short diffusion length can also lead to strong endurance performance, but further optimization work is required to investigate this.

## 4 Conclusion

In this paper we demonstrate unique resistive switching properties of amorphous YBCO thin films with different Cu contents in excess of stoichiometry. We demonstrate that resistive switching in these films can be tuned between a combined bulk- and interface-limited mechanism for 5% and 15% added Cu in the target, a filamentary mechanism for 25% added Cu, and an interfacial mechanism for 35% and 50% added Cu. Promising performance of an on/off ratio >100, switching endurance potential >6500 cycles, and state retention >2 × 10^4^ s was demonstrated with low switching fields ≤0.3 MV/cm in a system with high potential for further optimization. These results of versatility to tune between different switching mechanisms, combined with fast room temperature growth, gives amorphous YBCO with excess Cu strong potential to be explored further for future memory devices.

## Supplementary Material



## Data Availability

The raw data supporting this manuscript is openly available in the University of Cambridge repository Apollo with the doi 10.17863/CAM.127846.

## Notes

X-ray diffraction spectra of all deposited films (Figure S1); multiple *IV* curves of devices with varying Cu contents and electrode areas (Figure S2); comparison of all investigated films with YBCO- and Cu-based RS literature (Table S1); calculation of Fermi level position in YBCO (Supporting Note 1); analytical fitting of Schottky emission and space-charge-limited conduction (Figure S3); *IV* curve transition from filamentary to area switching (Figure S4); elemental EDX maps of the prepared samples (Figure S5); high-magnification transmission electron micrograph of clusters in YBCO (Figure S6); XPS scans of the prepared samples (Figure S7); cation ratio quantification from XPS (Table S2) (PDF)
