# Supplementary material for: Tunable Interfacial to Filamentary Resistive Switching Mechanism in Room-Temperature-Grown Amorphous YBa2Cu3O x with Excess Cu Addition
Source: ACS Appl Mater Interfaces. 2026 Jul 7;18(28):38982–91. doi: 10.1021/acsami.5c23772 (PMC13397493; doi:10.1021/acsami.5c23772)
Supplement: Supplementary file 1 [file am5c23772_si_001.pdf]

Supporting Information

Tuneable interfacial to filamentary resistive switching mechanism in room-temperature-grown amorphous $YBa_2Cu_3O_x$ with excess Cu addition

John Feighan[1], *Ingon Kim*[1], *Ahmed Kursumovic*[1], *Tamsin Bedford*[1], *Abhijeet Choudhury*[2], *Juanjuan Lu*[2], *Sebastian C. Dixon*[1], *Haiyan. Wang*[2], *Judith L. MacManus Driscoll*[1,]*, *Markus Hellenbrand*[1,]*

[1]Department of Materials Science & Metallurgy, University of Cambridge, 27 Charles Babbage Road, Cambridge CB3 0FS, United Kingdom

[1]School of Materials Engineering, Purdue University, Neil Armstrong Hall of Engineering, 701 West Stadium Avenue, IN 47907-2045, USA

*Corresponding authors: jld35@cam.ac.uk, mkhh2@cam.ac.uk

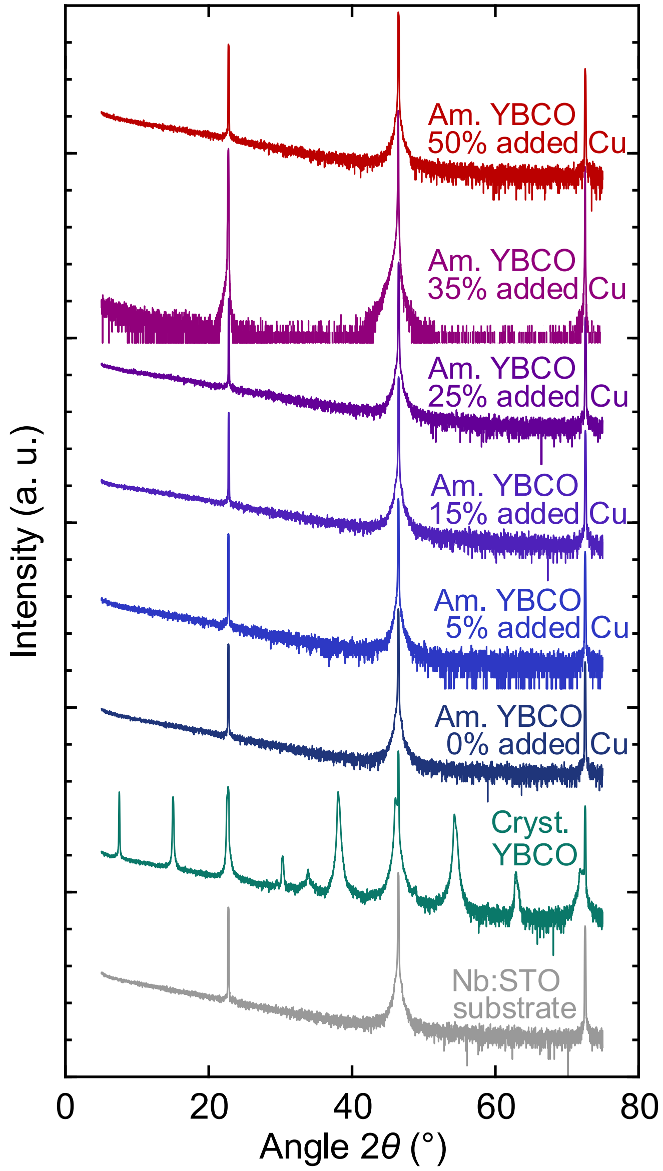

Figure S1: X-ray diffraction spectra for all samples in this study, plus reference spectra for crystalline YBCO and the Nb:STO substrate. The peaks of the latter are visible in all spectra. Other than these substrate peaks, none of the samples in this study show any peaks, confirming that there is no long-range crystallinity present, and the YBCO is amorphous. The 35% sample has a different background than the other samples, as it was measured with a different detector due to instrument availability.

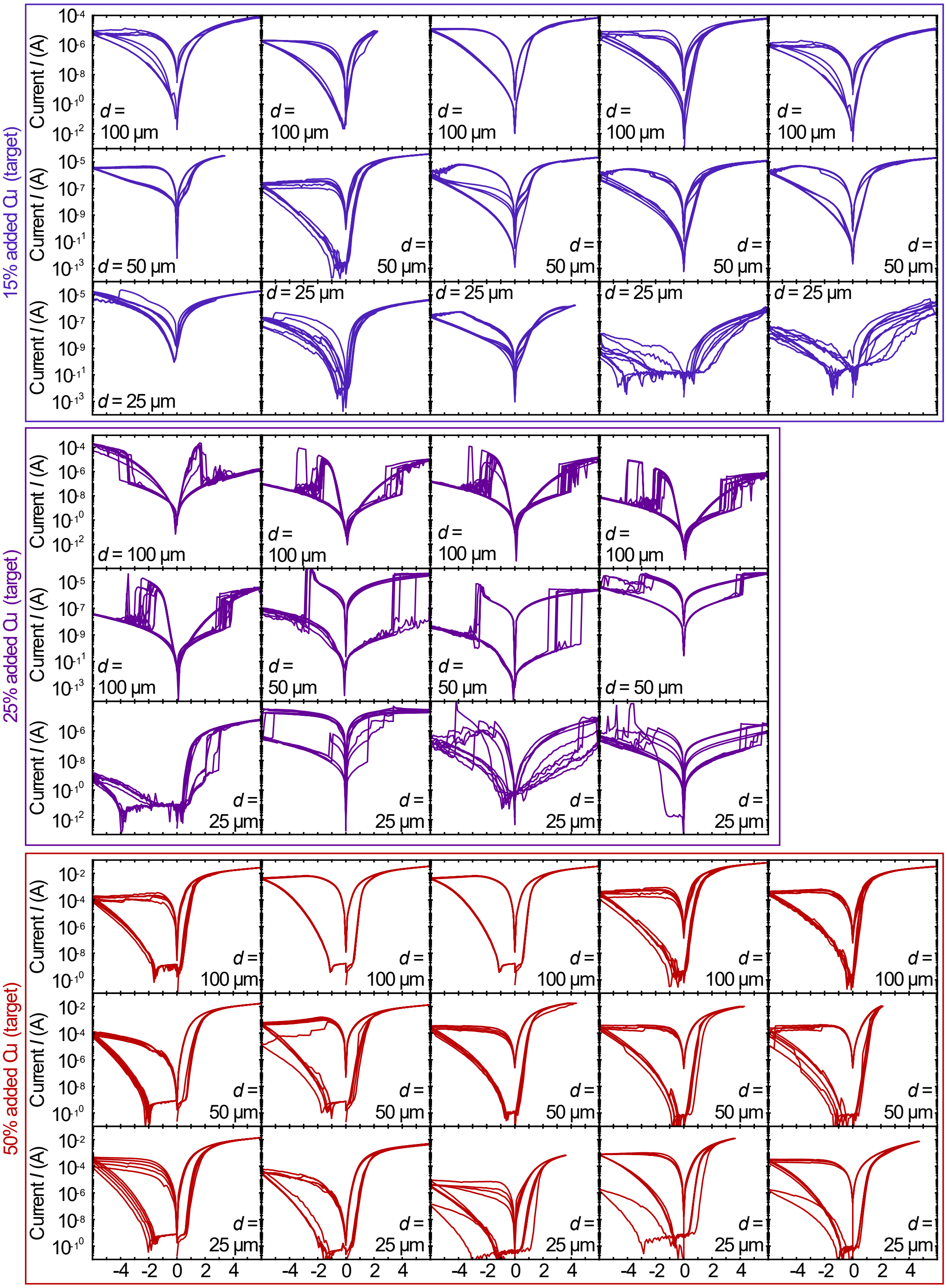

Figure S2: Example *IV* curves of the different switching mechanisms. (a) YBCO area switching, (b) filamentary switching, (c) YBCO/Nb:STO interface switching. These *IV* curves were also used to extract the area dependence. Note that while the variability increases with decreasing device diameter, qualitatively, the switching sequence remains the same.

Table S1: Comparison of two-terminal YBCO and Cu-based thin film resistive switching devices in the literature and various Cu concentrations from this work. Note that several publications exist with four-probe YBCO resistive switching devices, but the geometry and type of reported data prevent comparison of typical switching metrics.

Materials system	Deposition temperature	Switching mechanism	Switching electric field (MV/cm)	ON/OFF ratio* at V_{read} ≤ 1 V (approximate, from *IV* curves)	Endurance (presented, in cycles)	Retention* (presented)	Reference
Au/YBCO/Nb:STO	750 °C	Electron trapping	0.13-0.33	100	100	<10 s	[1]
Ag/YBCO/$LaAlO_3$	815 °C	Oxygen vacancy movement	0.03-0.2	>10**	-	-	[2]
YBCO/Nb:STO/YBCO, YBCO/Nb:STO/In	800 °C	Electron trapping	0.2	?**	-	-	[3]
Cu/YBCO/Nb:STO, 5% added Cu	RT	Electron trapping and oxygen (vacancy) movement	0.3	40	-	>10^4 s	This work
Cu/YBCO/Nb:STO, 15% added Cu	RT	Electron trapping and oxygen (vacancy) movement	0.3	10^3	>6500	>10^4 s	This work
Cu/YBCO/Nb:STO, 25% added Cu	RT	Cu filament	0.1	900	-	>10^3 s	This work
Cu/YBCO/Nb:STO, 35% added Cu	RT	Electron trapping	0.3	10^5	-	>10^4 s	This work
Cu/YBCO/Nb:STO, 50% added Cu	RT	Electron trapping	0.3	10^5	-	>10^4 s	This work
Au-Pd/CuO_2/Au	60 °C	Cu filament	0.004-0.009***	10^3	170	-	[4]
Cu/polymer/ITO	?	Cu filament	0.075-0.2	30	50	>300 s	[5]
Cu/TaOx/TiON/TiN	RT/550 °C	Cu + V_O filament	0.5	40	200	-	[6]
Cu-TiW/IGZO/Al_2O_3/Pt	RT?	Cu filament	1-1.5	10^6	10^3	10^3 s	[7]
Cu/TiW/Al_2O_3/W	300/400 °C	Cu filament	6.7	10^3	>10^6	Hours	[8]

*These values were extracted for measurements at room temperature or higher to be able to compare between all reports.
**Data were only presented on linear current axes, which makes this estimation very approximate or impossible.
***Note that while the switching field is low, the devices used 5 μm thick films and needed a forming voltage of 34 V.

Supporting Note 1: Position of the Fermi level in amorphous YBCO

As mentioned in the main text, YBCO is typically a p-type material, and its hole carrier concentration can be estimated as $p < 10^{22}$ cm^{-3} for its amorphous state [9]. The position of the Fermi level E_F with respect to the valence band edge E_V can then be calculated as [10]

$$E_F - E_V = k_B T \ln\left(\frac{N_V}{p}\right), \tag{S1}$$

where k_B is the Boltzmann constant, T the temperature, and N_V is the valence band effective density of states, which itself can be calculated as [10]

$$N_V = 2\left(\frac{2\pi m_h^* k_B T}{h^2}\right)^{3/2}, \tag{S2}$$

where m_h^* is the effective hole mass, h is Planck's constant, and the other variables as before.

With $p = 10^{22}$ cm^{-3} (as an example value for $p < 10^{22}$ cm^{-3}) and the only other literature value $m_h^* = 2m_0$ (m_0 being the electron rest mass) [11], $E_F - E_V = -0.13$ eV, i.e. the Fermi level rests slightly below the valence band edge. For $p = 10^{21}$ cm^{-3} as an example of $p < 10^{22}$ cm^{-3}, $E_F - E_V = -0.07$ eV, and for smaller p, the Fermi level will eventually move into the band gap for $p < N_V$.

It is noted that more accurate expressions exist when the Femi level moves out of the band gap [10], but for the present estimation, (S1) should be sufficient to construct a band diagram and explain the switching mechanism.

The band diagram in the main Figure 2(i) reflects a situation where E_F is still slightly inside the valence band. Importantly, the exact value of the hole concentration does not have a large impact on the barrier height between the YBCO and the substrate or top electrode.

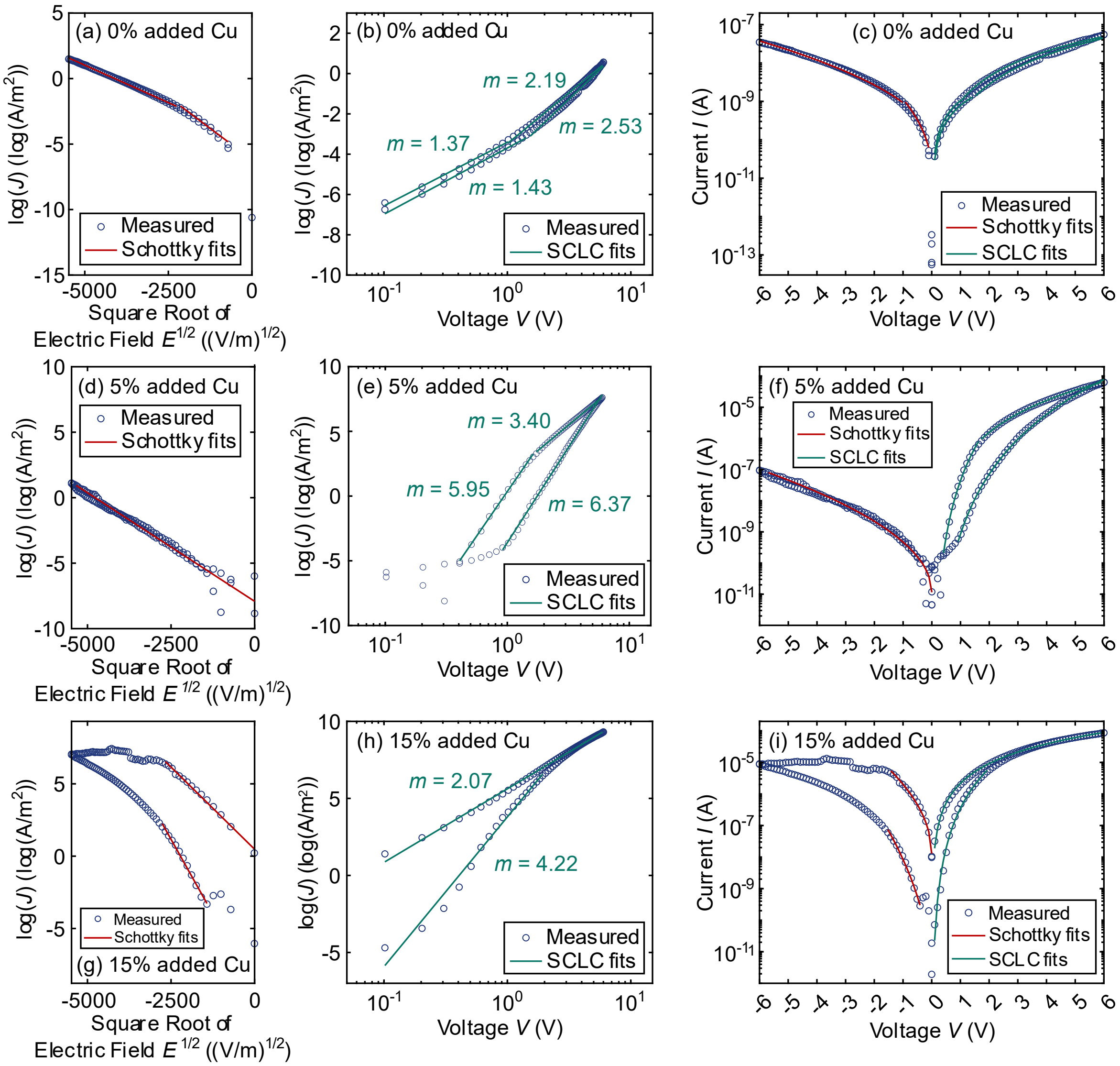

Figure S3.1: Measured *IV* curves fitted with electronic conduction models for added Cu concentrations of 0-15%. Remaining added Cu concentration continued in Figure S2.2. Based on the band diagram in main Figure 2(g), the forward direction (positive voltage at Cu top electrode) can be expected to be governed by space-charge-limited conduction (SCLC), and the reverse direction (negative voltage at Cu top electrode) by a barrier-limited transport mechanism such as Schottky emission. Indeed, these models fit the measured data reasonably well. The left panels show the reverse direction Schottky fits, the centre panels show the forward direction SCLC fits, and the right panels shows the fits overlayed on the measured *IV* curves. The conduction mechanisms change with the Cu content, as explained in the main text.

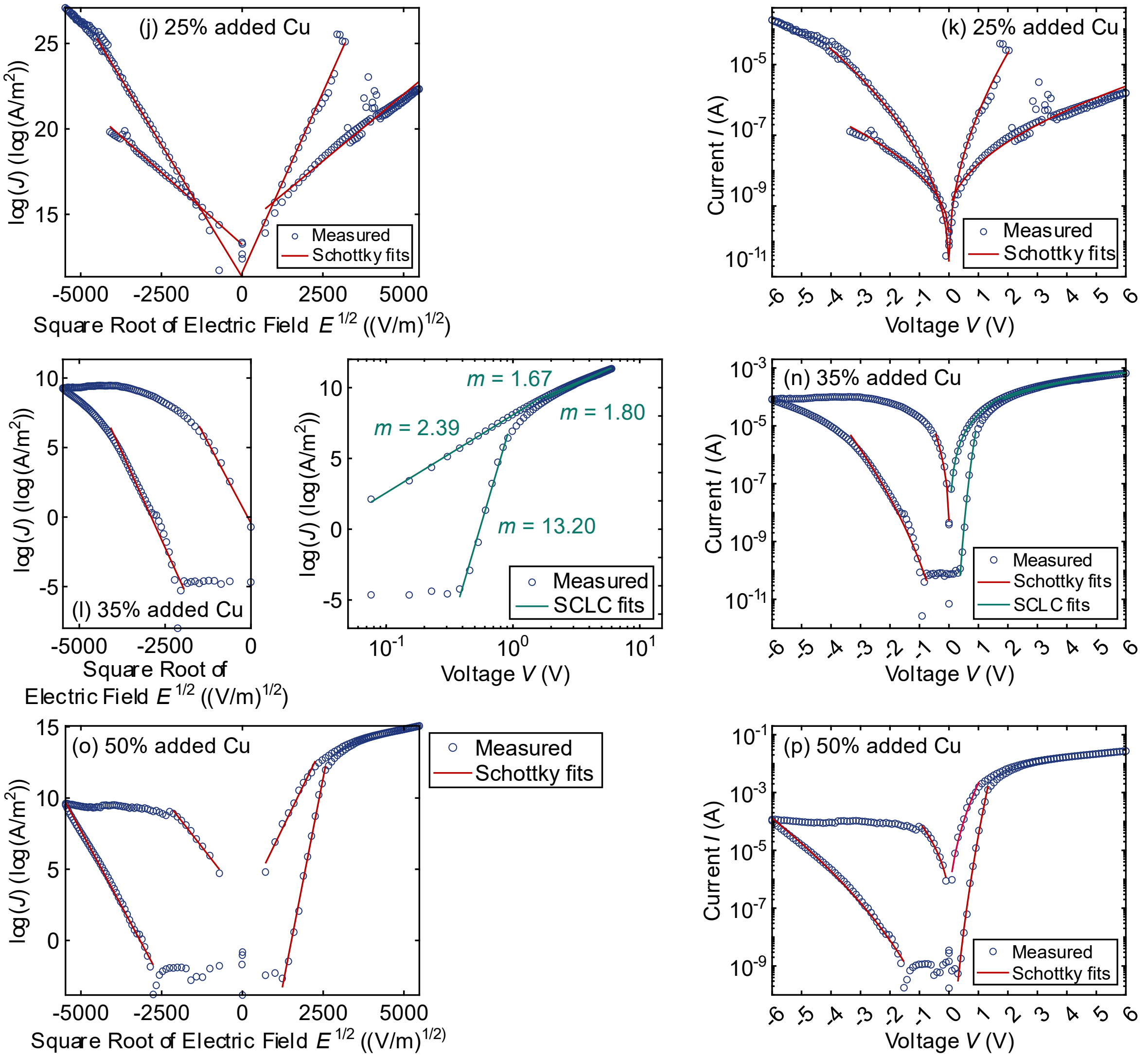

Figure S3.2: Measured *IV* curves fitted with electronic conduction models for added Cu concentrations of 25-50%; continuation of Figure S2.1. For 35% added Cu, the reverse/forward separation still applies, for the filamentary 25% and the fully interfacial 50% added Cu, only Schottky emission fits the data well.

For Schottky emission, the standard textbook equation

$$J_{\mathrm{SE}} = A^{*}T^{2}\exp\left[\frac{q}{k_{\mathrm{B}}T}\left(\sqrt{\frac{qV}{4\pi\epsilon_{\mathrm{op}}\epsilon_{0}t_{\mathrm{ox}}}}-\Phi_{\mathrm{B}}\right)\right] \tag{S3}$$

was fitted to the data, where J_{SE} is the measured current density, $A^{*} = \lambda A_0$ is the effective Richardson constant, where $A_0 = 1.2\times10^{6}$ Am^{-2}K^{-2} and λ is typically of the order 0.5 [12], $T = 291$ K is the measurement temperature, q the elemental charge, k_{B} the Boltzmann constant, V the applied voltage, ϵ_{op} the optical

dielectric constant of the thin film, ϵ_0 the vacuum permittivity, t_{ox} the thin film thickness, and Φ_B the interface barrier height, which can be calculated from the fitted data.

For samples with area scaling, the entire device area was used to calculate the current density for fitting. For the filamentary sample (25% added Cu), a circular area with a diameter of 10 nm was assumed to represent the filament. As evident from the equation, the film thickness and the dielectric constant are not required to calculate the barrier height. This is convenient because the effective thickness over which the voltage drops is a significant source of uncertainty. The nominal film YBCO film thickness is 200 nm, but in the case of filamentary switching (25% added Cu) or conductive YBCO (35-50% added Cu), the majority of the voltage can be expected to drop in a narrow region near the interface to the Nb:STO substrate. This affects very strongly the second free parameter in the fitting exercise – the optical dielectric constant. While YBCO can exhibit very high dielectric constants at low frequencies, it falls off rapidly towards optical frequencies. When varying the thickness in the Schottky equation between 5 nm and 200 nm, the fitted dielectric constats vary between about 40 and slightly below 1. Most reports on the dielectric constant of YBCO focus on the low-frequency range, so it is difficult to evaluate whether the fitted values confirm the validity of the assumed conduction model. For reference of the x-axes in Figure S2, all plots are made with 200 nm oxide thickness for figure uniformity.

As to the thickness-independent calculation of the Schottky barrier height, for the added Cu concentrations of 0-15%, the calculated values from the fits in the negative voltage ranges are between 0.61 and 0.85 eV for the low resistance (LRS) and high resistance state (HRS), respectively. For the filamentary devices with 25% added Cu, the barrier height is ≈0.24 eV in the LRS and ≈0.30 eV in the HRS. For the samples with 35% and 50% added Cu, the Schottky barrier heights are between 0.54 eV and 1.26 eV for the LRS and HRS, respectively. This constitutes a much higher HRS barrier value than for the lower-Cu-content samples, but the values are in line with previous reports of metal electrodes directly on Nb:STO[13], which supports the understanding that here, in the samples 35-50% added Cu, the YBCO forms a very conductive layer on top of the Nb:STO and mostly just acts as an electrode.

For SCLC, only the slopes of the current density vs. the voltage in double-logarithmic plots were fitted to identify the potential mechanism and the different areas of conduction limitation, such as the amount of trap filling. Higher slopes indicate strong trap-filling-affected conduction, whereas lower slopes indicate mostly filled traps[14].

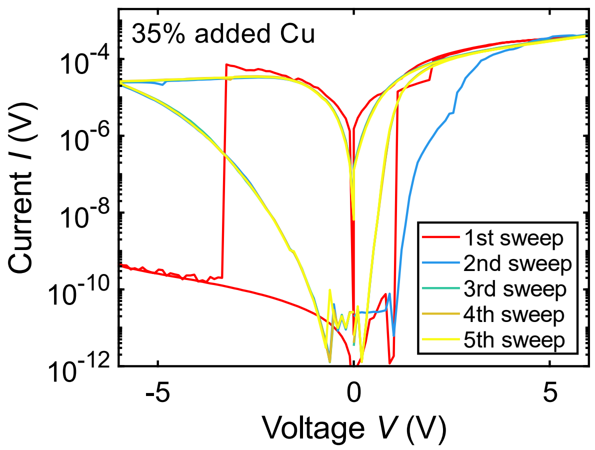

Figure S4: Initial five *IV* curves for a device with 35% added Cu. For some of these, the *IV* curve initially exhibited filamentary switching (red), before gradually (blue) changing to non-filamentary switching (green-yellow), where the *IV* hysteresis is determined by charge trapping at the YBCO/Nb:STO interface.

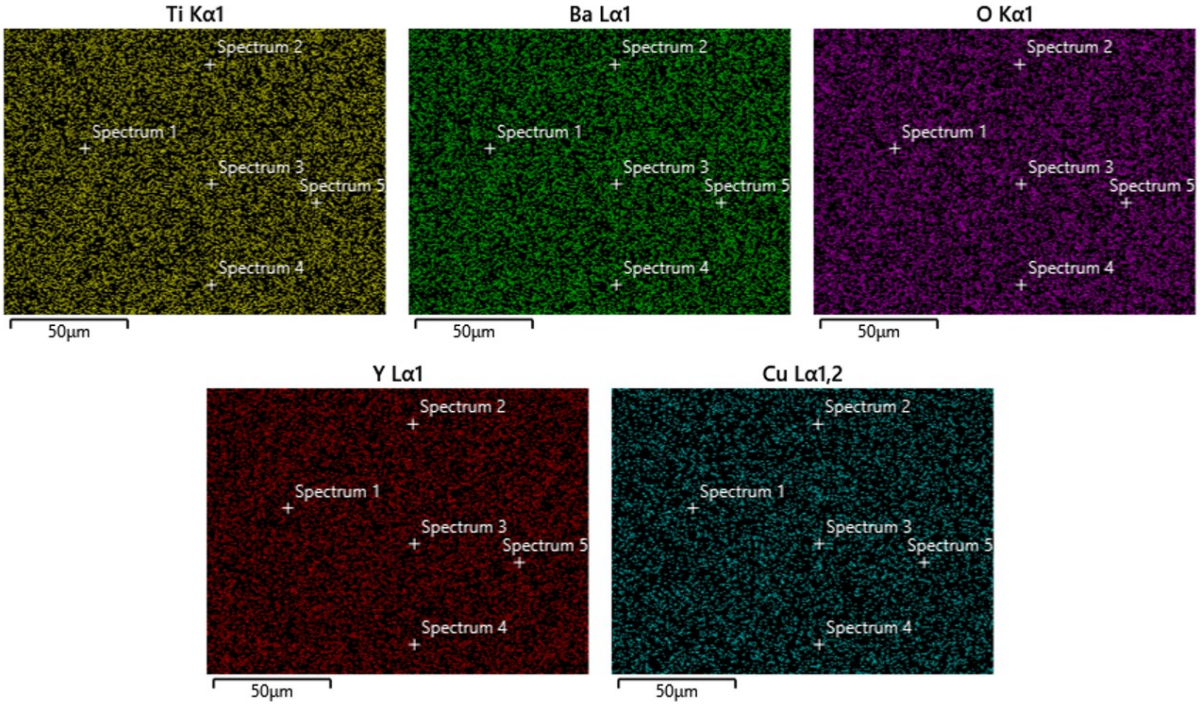

Figure S5.1: EDX elemental maps for the 0% added Cu sample. Labelled spectra indicate locations selected to compare the bulk composition with local environments. The averages of all spot measurements are summarised in Supplementary Table S2.

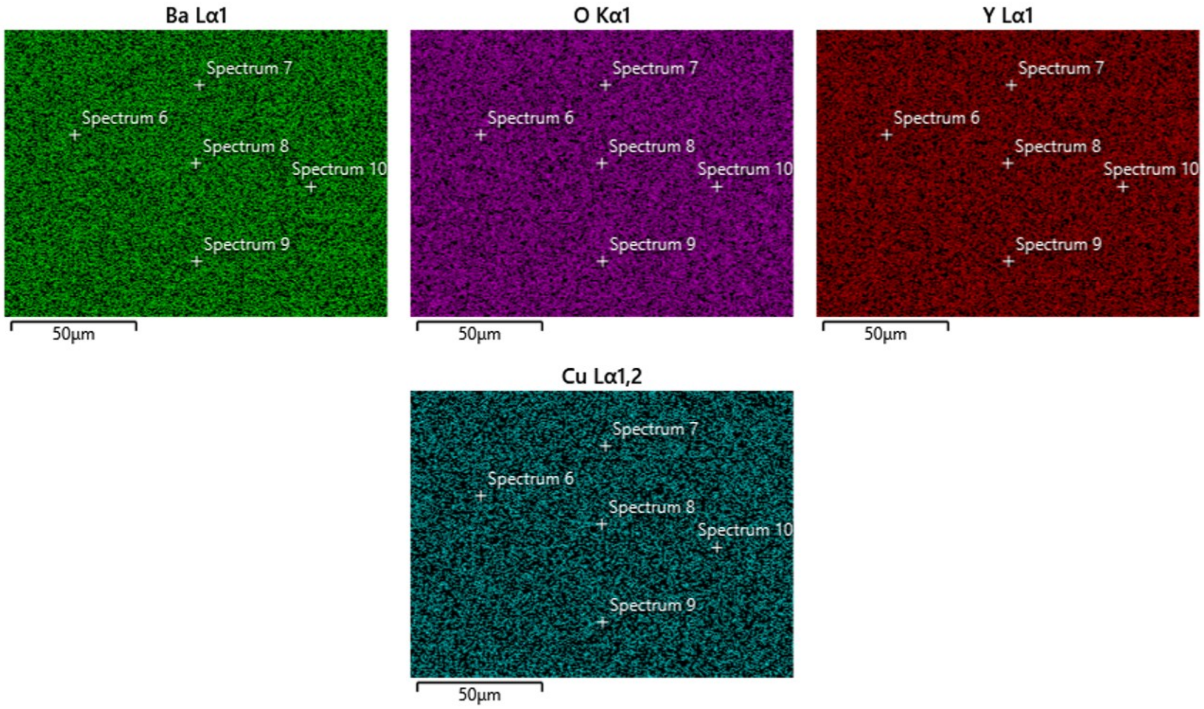

Figure S5.2: EDX elemental maps for the 5% added Cu sample. Labelled spectra indicate locations selected to compare the bulk composition with local environments. The averages of all spot measurements are summarised in Supplementary Table S2.

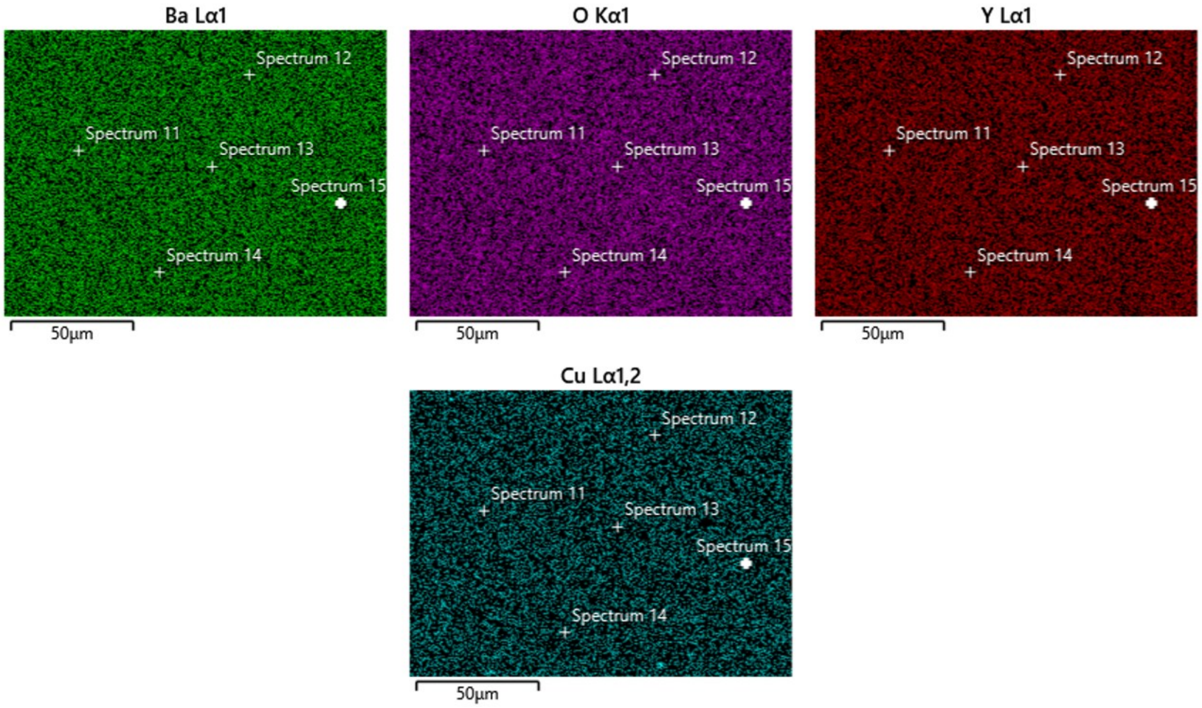

Figure S5.3: EDX elemental maps for the 15% added Cu sample. Labelled spectra indicate locations selected to compare the bulk composition with local environments. The averages of all spot measurements are summarised in Supplementary Table S2.

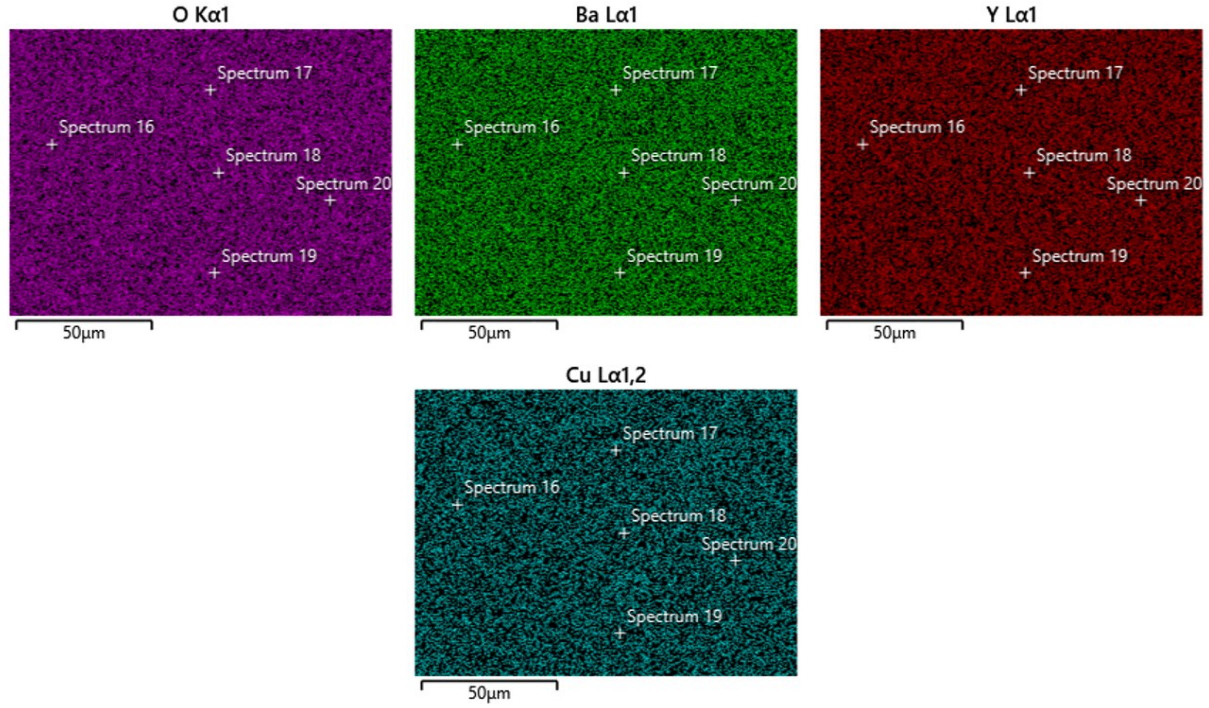

Figure S5.4: EDX elemental maps for the 25% added Cu sample. Labelled spectra indicate locations selected to compare the bulk composition with local environments. The averages of all spot measurements are summarised in Supplementary Table S2.

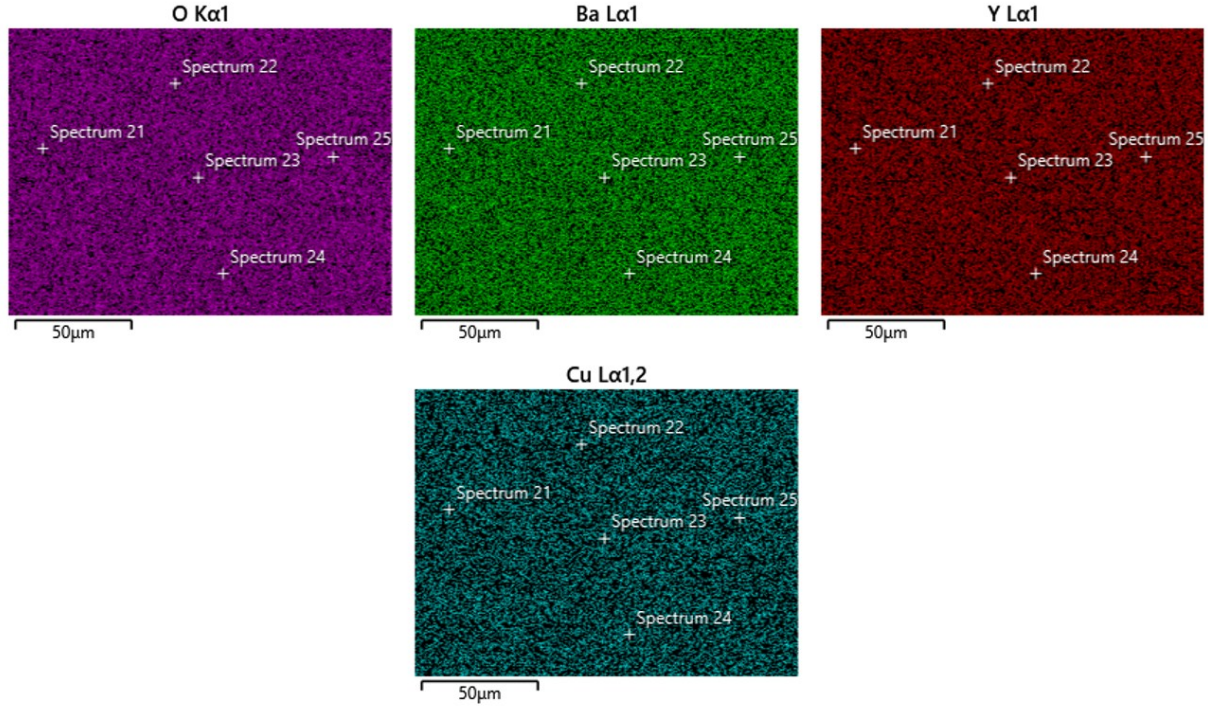

Figure S5.5: EDX elemental maps for the 35% added Cu sample. Labelled spectra indicate locations selected to compare the bulk composition with local environments. The averages of all spot measurements are summarised in Supplementary Table S2.

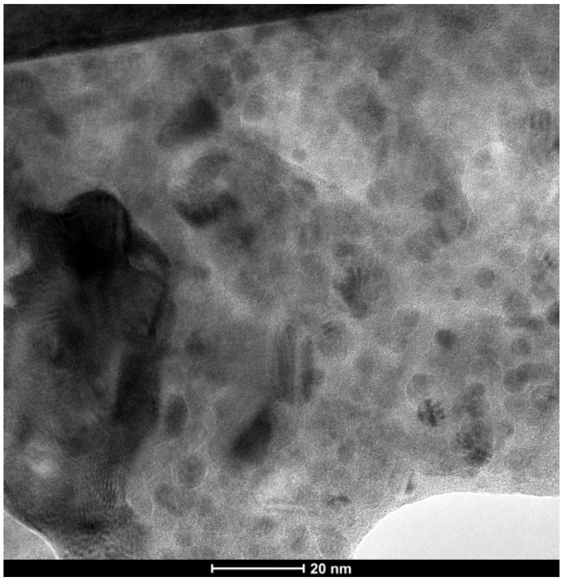

Figure S6: High magnification transmission electron micrograph of clusters in a film deposited with 50% added Cu in the target. Note that the image is ‘upside down’ due to target preparation, i.e. the Nb:STO substrate is at the top of the image.

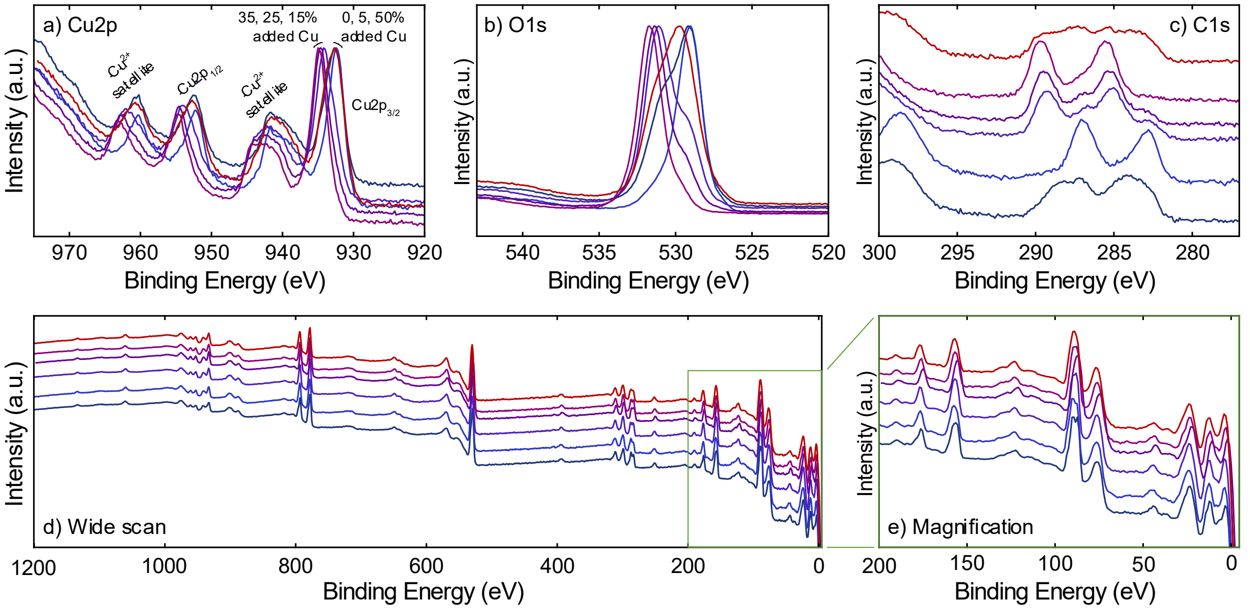

Figure S7: X-ray photoelectron spectroscopy (XPS) scans of all samples. The PLD target added Cu concentration increases from blue to red curves. a) Cu2p spectrum. The marked satellites are the hallmark of Cu^{2+}; the Cu^{1+} satellite would appear as a weak signal at about 945 eV and cannot be detected here. Due to the lack of a reliable reference standard in this measurement (the instrument uses Cu sample holders), the peak position cannot be corrected reliably. b) O1s peaks. For the same reason of missing a reliable standard in this measurement, the peak positions could not be corrected. c) The complicated C1s peak indicates various species of carbonaceous adsorbates. d) Wide spectrum of all films. e) Magnified region of the lowest binding energies.

Table S2: Cation ratio quantification from XPS. The Y3d, Cu2p, and Ba3d peaks were used. The resulting ratios are much lower than both the EDX results and the target composition, which can be explained by preferential Cu sputter etching due to its relatively lowest mass among the present cations. However, given that the Ba/Y ratio from XPS is similar to the EDX results, the contribution of potential yttrium carbonates seems negligible. The EDX results are copied from main text Table 1 for reference.

Added Cu	PLD target, nominal	Cu/Y ratio EDX area	Cu/Y ratio EDX spots	Cu/Y ratio XPS wide	Ba/Y ratio EDX area	Ba/Y ratio EDX spots	Ba/Y ratio XPS area
0%	$Y_1Ba_2Cu_3O_x$	3.93	4.04	1.69	1.64	1.64	1.65
5%	$Y_1Ba_2Cu_{3.15}O_x$	4.44	4.72	2.24	1.38	1.66	1.81
15%	$Y_1Ba_2Cu_{3.45}O_x$	4.90	4.89	1.93	1.70	1.6	1.70
25%	$Y_1Ba_2Cu_{3.75}O_x$	5.23	5.14	2.06	1.46	1.59	1.59
35%	$Y_1Ba_2Cu_{4.05}O_x$	6.29	6.95	2.70	1.71	1.81	1.71

References

1. Zhang, H. J., Zhang, X. P., Shi, J. P., Tian, H. F. & Zhao, Y. G. Effect of oxygen content and superconductivity on the nonvolatile resistive switching in $YBa_2Cu_3O_{6+x}$/Nb-doped $SrTiO_3$ heterojunctions. *Applied Physics Letters* **94**, 092111 (2009) https://doi.org/10.1063/1.3095493.
2. Plecenik, A., Tomasek, M., Plecenik, T., Truchly, M., Noskovic, J., Zahoran, M., Roch, T., Belogolovskii, M., Spankova, M., Chromik, S., *et al.* Studies of resistance switching effects in metal/$YBa_2Cu_3O_{7-x}$ interface junctions. *Applied Surface Science* **256**, 5684–5687 (2010) https://doi.org/10.1016/j.apsusc.2010.03.018.
3. Jia, J., Gao, J., He, Y., Zhao, G. & Ren, Y. Resistive switching effect of $YBa_2Cu_3O_{7-x}$/Nb:$SrTiO_3$ heterostructure. *Materials Research Bulletin* **107**, 328–332 (2018) https://doi.org/10.1016/j.materresbull.2018.08.005.
4. Yazdanparast, S., Koza, J. A. & Switzer, J. A. Copper Nanofilament Formation during Unipolar Resistance Switching of Electrodeposited Cuprous Oxide. *Chem. Mater.* **27**, 5974–5981 (2015) https://doi.org/10.1021/acs.chemmater.5b02041.
5. Kharlanov, O. G., Shvetsov, B. S., Rylkov, V. V. & Minnekhanov, A. A. Stability of Quantized Conductance Levels in Memristors with Copper Filaments: Toward Understanding the Mechanisms of Resistive Switching. *Phys. Rev. Appl.* **17**, 054035 (2022) https://doi.org/10.1103/PhysRevApplied.17.054035.
6. Jeon, H., Park, J., Jang, W., Kim, H., Song, H., Kim, H., Seo, H. & Jeon, H. Resistive switching behaviors of Cu/TaO_x/TiN device with combined oxygen vacancy/copper conductive filaments. *Current Applied Physics* **15**, 1005–1009 (2015) https://doi.org/10.1016/j.cap.2015.06.002.
7. Gan, K.-J., Chang, W.-C., Liu, P.-T. & Sze, S. M. Investigation of resistive switching in copper/InGaZnO/Al_2O_3-based memristor. *Appl. Phys. Lett.* **115**, 143501 (2019) https://doi.org/10.1063/1.5116359.
8. Belmonte, A., Kim, W., Chan, B., Heylen, N., Fantini, A., Houssa, M., Jurczak, M. & Goux, L. 90nm W\Al_2O_3\TiW\Cu 1T1R CBRAM cell showing low-power, fast and disturb-free operation. in *2013 5th IEEE International Memory Workshop* 26–29 (2013). https://doi.org/10.1109/IMW.2013.6582089.
9. Okunev, V. D., Samoilenko, Z. A., Svistunov, V. M., Abal'oshev, A., Dynowska, E., Gierłowski, P., Klimov, A. & Lewandowski, S. J. Amorphous state and pulsed laser deposition of $YBa_2Cu_3O_{7-\delta}$ thin films. *J. Appl. Phys.* **85**, 7282–7290 (1999) https://doi.org/10.1063/1.370545.
10. Sze, S. M. & Ng, K. K. Semiconductor Physics. in *Physics of Semiconductor Devices* (John Wiley & Sons, Inc., Hoboken, New Jersey, 2007).
11. Padilla, W. J., Lee, Y. S., Dumm, M., Blumberg, G., Ono, S., Segawa, K., Komiya, S., Ando, Y. & Basov, D. N. Constant effective mass across the phase diagram of high-T_c cuprates. *Phys. Rev. B* **72**, 060511 (2005) https://doi.org/10.1103/PhysRevB.72.060511.
12. Crowell, C. R. The Richardson constant for thermionic emission in Schottky barrier diodes. *Solid-State Electronics* **8**, 395–399 (1965) https://doi.org/10.1016/0038-1101(65)90116-4.
13. Mikheev, E., Hoskins, B. D., Strukov, D. B. & Stemmer, S. Resistive switching and its suppression in Pt/Nb:$SrTiO_3$ junctions. *Nat Commun* **5**, 3990 (2014) https://doi.org/10.1038/ncomms4990.
14. Lampert, M. A. Simplified Theory of Space-Charge-Limited Currents in an Insulator with Traps. *Phys. Rev.* **103**, 1648–1656 (1956) https://doi.org/10.1103/PhysRev.103.1648.
